# Soybean β-Conglycinin and Cowpea β-Vignin Peptides Inhibit Breast and Prostate Cancer Cell Growth: An In Silico and In Vitro Approach

**DOI:** 10.3390/foods13213508

**Published:** 2024-11-01

**Authors:** Biane Oliveira Philadelpho, Victória Guimarães Santiago, Johnnie Elton Machado dos Santos, Mariana Barros de Cerqueira e Silva, Rone Aparecido De Grandis, Eduardo Maffud Cilli, Fernando Rogério Pavan, Marcelo Santos Castilho, Alessio Scarafoni, Carolina Oliveira de Souza, Ederlan de Souza Ferreira

**Affiliations:** 1 School of Pharmacy, Federal University of Bahia, Barão de Jeremoabo Street, Salvador 40170-115, BA, Brazil; biane_philadelpho@hotmail.com (B.O.P.); victoria.santiago@hotmail.com (V.G.S.); johnnie.machado25@gmail.com (J.E.M.d.S.); castilho@ufba.br (M.S.C.); carolods@ufba.br (C.O.d.S.); 2 School of Pharmacy, Federal University of Amazonas, General Rodrigo Octávio Jordão Ramos Avenue, Manaus 69067-005, AM, Brazil; marianabarros@ufam.edu.br; 3 School of Pharmacy, São Paulo State University (UNESP), Araraquara-Jaú Road, Araraquara 14800-903, SP, Brazil; degrandis.rone@gmail.com (R.A.D.G.); fernando.pavan@unesp.br (F.R.P.); 4 Institute of Chemistry, São Paulo State University (UNESP), Prof. Francisco Swgni Street, Araraquara 14800-060, SP, Brazil; eduardo.cilli@unesp.br; 5 Department of Food, Environmental and Nutritional Sciences (DeFENS), Università degli Studi di Milano, 20133 Milan, Italy; alessio.scarafoni@unimi.it

**Keywords:** leguminous bioactive peptides, cell viability, docking, structure–activity relationships, Bcl-2, VIPAAY peptide, BH3-mimetic effect

## Abstract

B-cell lymphoma 2 protein (Bcl-2) is an important regulator of cell apoptosis. Inhibitors that mirror the structural domain 3 (BH3) of Bcl-2 can activate apoptosis in cancer cells, making them a promising target for anticancer treatment. Hence, the present study aimed to investigate potential BH3-mimetic peptides from two vicilin-derived legume proteins from soybean and cowpea bean. The proteins were isolated and sequentially hydrolyzed with pepsin/pancreatin. Peptides < 3 kDa from vicilin-derived proteins from soybean and cowpea beans experimentally inhibited the growth of cultivated breast and prostate cancer cells. In silico analysis allowed the identification of six potential candidates, all predicted to be able to interact with the BH3 domain. The VIPAAY peptide from the soybean β-conglycinin β subunit showed the highest potential to interact with Bcl-2, comparable to Venetoclax, a well-known anticancer drug. Further experiments are needed to confirm this study’s findings.

## 1. Introduction

Legumes play a crucial role in the dietary habits of numerous populations across the globe due to their nutritional profile, as they are a rich source of fiber, proteins, vitamins, and minerals [1]. They are cultivated in an environmentally sustainable way, making them an economical source of proteins compared to those of animal-derived origins [2]. Besides their nutritional value, their consumption displays a variety of well-documented health benefits [3] attributable to their bioactive compounds, such as polyphenols, isoflavones, phytosterols, lectins, and peptides [4]. The increased consumption of legumes has been demonstrated to mitigate/prevent diseases [5]. Thus, the potential of legumes as a natural source of bioactive compounds has been extensively studied, particularly peptides derived from their proteins [6,7].

Bioactive peptides derived from the storage protein of legumes have been related to several biological properties, including hypocholesterolemic [8,9,10], antioxidant [11], antimicrobial [12], and anticancer activity [7], but the mechanism exerted by these peptides has not been completely elucidated. On the other hand, the anticancer effects in colon cancer and leukemic cells have been attributed to peptides derived from soybean protein. The lunasin peptide has been highlighted as a potent epigenetic modulator by inhibiting histone acetylation, inducing apoptosis, and regulating the cell cycle of cancer cells [13].

Recently, anticancer peptides have emerged as a promising therapeutic strategy for cancer treatment since they have high specificity and selectivity, low toxicity, and the ability to target “undruggable” proteins [14]. B-cell lymphoma 2 protein (Bcl-2) is considered an “undruggable protein” due to its lack of defined ligand binding pockets, non-catalytic protein–protein interactions, and less-explored 3D structures [15]. The Bcl-2 family of proteins comprises apoptotic-related proteins involved in the intrinsic (or mitochondrial) pathway [16]. Anti-apoptotic proteins include the multi-BH domain proteins Bcl-2, Bcl-w, Bcl-xL, Mcl-1, and A1, whereas pro-apoptotic proteins include the multi-BH domain effector proteins (Bax and Bak) and BH3-only proteins (Bad, Bim, Bid, Bik, Bmf, Hrk, Noxa, and Puma). BH3-only proteins can bind to the BH3 domain of anti-apoptotic proteins (such as Bcl-2) when the cell detects a death signal. Therefore, Bcl-2 loses its ability to inhibit the apoptotic pathway since its BH3 domain cannot interact with its pro-apoptotic counterparts [17]. Bcl-2 is estimated to be overexpressed in several human cancers, such as leukemia [18], colorectal carcinoma [19], breast cancer [20], and prostate cancer [21], among others.

In the present study, we investigated the anticancer effects of peptides derived from soybean β-conglycinin and cowpea β-vignin proteins following in vitro digestion with pepsin/pancreatin, using breast and prostate cancer model cells. To identify candidate peptides that theoretically originated after proteolysis and could be involved in the observed biological effects, we then performed bioinformatic studies focusing on their potential ability to interact with the BH3 domain of the Bcl-2 protein to delineate a possible mechanism of action.

## 2. Materials and Methods

### 2.1. Plant Material and Reagents

The seeds of soybean (*Glycine max*) and cowpea bean (*Vigna unguiculata*) were obtained from the northeast region of the State of Bahia, kindly provided by the Bahiana Agricultural Development Company. The non-cancer endothelial cell line from the umbilical cord (HUVEC—ATCC CRL-1730^TM^), the cancer cells from human breast adenocarcinoma (MDA-MB-231—ATCC HTB-26^TM^), and prostate carcinoma cells (DU-145—ATCC HTB-81^TM^) were obtained from the American Type Cell Collection (ATCC) (Manassas, VA, USA). Bovine serum albumin (CAS 9048-46-8), sodium dodecyl sulfate (CAS n° 151-21-3), Coomassie brilliant blue (CAS n° 6104-59-2), methyl methanesulfonate (CAS n° 66-27-3), dimethyl sulfoxide (CAS n° 67-68-5), resazurin (CAS n° 62758-13-8), pepsin (CAS n° 9001-75-6), and pancreatin (CAS n° 8049-47-6) enzymes were purchased from Sigma-Aldrich^®^ (St. Louis, MO, USA). All reagents were purchased from Sigma-Aldrich^®^ unless otherwise specified.

### 2.2. Separation and Isolation of β-Conglycinin and β-Vignin

Initially, the cotyledon was separated from the grains, dehydrated, crushed, and sieved through a 60-mesh screen [8]. The whole soy flour was defatted, following established procedures [22]. The defatted soy flour and whole cowpea flour were stored in a polyethylene container and kept refrigerated at 4 °C. The β-conglycinin and β-vignin proteins were isolated according to previous studies [22,23]. A brief description of the two procedures is outlined below.

The defatted soybean flour was suspended with water (1:15 *w*/*v* flour/water ratio), pH-adjusted to 7.5, and centrifuged (2000× *g* for 30 min). The supernatant was treated with sodium bisulfite (0.98 g/L), and the pH was adjusted to 6.4. The solution was stirred at 4 °C overnight and centrifuged (6500× *g* for 20 min). The soluble fraction was adjusted to pH 5.0 with 0.25 mol/L NaCl and, after 1 h of gentle stirring at 4 °C, centrifuged (9000× *g* for 30 min). The precipitate fraction containing the β-conglycinin protein was finally solubilized in 0.25 mol/L sodium phosphate buffer (pH 7.0) containing 0·2 mol/L NaCl, before undergoing gel filtration chromatography.

To obtain the β-vignin protein, the cowpea flour was suspended with 0.1 mol/L NaCl (1:20 *w*/*v* flour), adjusted to 7.5 pH, stirred for 1 h at room temperature, and centrifuged (10,000× *g* for 40 min). The supernatant was kept at 4 °C. The pellet was dissolved in water (1:20 w (initial weight)/v), the pH was adjusted to 7.5, and the mixture was stirred at 4 °C for 10 min before being centrifuged as described previously. The two supernatants were pooled and diluted 1/1 (*v*/*v*) with distilled water, and the pH was adjusted to 5.0 with diluted HCl and kept at 4 °C overnight. After centrifugation (10,000× *g* for 40 min), the insoluble fraction containing β-vignin was dissolved in 0.25 mol/L sodium phosphate buffer (pH 7.0) containing 0.2 mol/L NaCl for subsequent subjection to gel filtration chromatography, as reported below.

Both proteins were then purified using size exclusion chromatography with a Sepharose CL-6B column (1.0 × 100 cm) in previously established conditions [8]. The elution profile was monitored by measuring the absorbance at 280 nm. The peak corresponding to the proteins was collected, dialyzed, and lyophilized [24]. The protein was quantified with the Lowry method, using bovine serum albumin as a standard [25].

### 2.3. Sodium Dodecyl Sulfate–Polyacrylamide Gel Electrophoresis (SDS-PAGE)

β-conglycinin and β-vignin were analyzed using SDS-PAGE in polyacrylamide gel (12 g/100 g) with sodium dodecyl sulfate (0.1 g/100 g) [26]. The gels were stained in a Coomassie brilliant blue solution (R-250) and destained with methanol/acetic acid/water (1:1:8 *v*/*v*/*v*). The images were digitized and analyzed using ImageJ software (Alpha Innotech^®^, San Leandro, CA, USA).

### 2.4. In Vitro Gastrointestinal Digestion and Ultrafiltration

Samples of the purified proteins were hydrolyzed sequentially using pepsin (1:66 E/S) and pancreatin (1:25 E/S) [27]. Briefly, both isolated proteins (200 mg) were hydrolyzed by pepsin (37 °C for 3 h, pH = 2) and then further treated with pancreatin (37 °C for 3 h, pH = 7.5). The reaction was stopped by immersing the samples in an ice bath, followed by centrifugation (15,000× *g* for 15 min). The β-conglycinin and β-vignin hydrolysates were filtered using a 30 kDa Microcon^®^ centrifugal filter (Merck Millipore, Germany) to remove the enzymes (pepsin and pancreatin). The hydrolysate used in this study was the filtrate composed of ≤30 kDa peptides. Part of the hydrolysate was then filtered using a 3 kDa filter (Microcon^®^ Centrifugal Filter, Merck Millipore, Darmstadt, Germany) to obtain peptides < 3 kDa.

### 2.5. Cell Viability Assay

The non-cancer endothelial cell line from the umbilical cord (HUVEC—ATCC CRL-1730^TM^), the cancer cells from human breast adenocarcinoma (MDA-MB-231—ATCC HTB-26^TM^), and the prostate carcinoma cells (DU-145—ATCC HTB-81^TM^) were used in cell viability experiments. The cell viability was quantified using an Alamar Blue assay [28,29]. The cells were seeded in 96-well plates (1.5 × 10^4^ cells/well). After 24 h, the hydrolysates and peptide fractions less than 3 kDa were added to the wells at concentrations ranging from 12.5 to 200 μg/mL. The positive control (PC) consisted of cells that received the culture medium and the cytotoxic agent methyl methanesulfonate (MMS, 300 µM in dimethyl sulfoxide (DMSO) 5 mL/L). The negative control (NC) consisted of cells that received the culture medium and DMSO (5 mL/L). After 24 h of incubation, 50 μL of Alamar Blue (0.01%, *w*/*v* resazurin) was added to each well, and the plates were incubated for 1 h at 37 °C in the dark [29]. Fluorescence reading was performed in a hybrid multi-mode microplate reader (SynergyTM H1, BioTek^®^, Agilent, Santa Clara, CA, USA) using excitation and emission filters at wavelengths of 560 and 590 nm, respectively.

### 2.6. In Silico Analysis

#### 2.6.1. Protein Sequences and Simulated Gastrointestinal Digestion

Primary sequences of vicilins from soybean (*Glycine max*) and cowpea bean (*Vigna unguiculata*) were obtained from the UniProtKB database (https://www.uniprot.org/, accessed on 12 February 2024) (Table S1). Simulated gastrointestinal digestion with pepsin (EC 3.4.23.1), trypsin (EC 3.4.21.4), and chymotrypsin (EC 3.4.21.1) was performed using the BIOPEP server^®^ (http://www.uwm.edu.pl/biochemia/index.php/en/biopep, accessed on 16 January 2024) [9]. The amino acid concentration from the hydrolysis-derived peptides was calculated using the ProtParam Tool^®^ (http://web.expasy.org/protparam/, accessed on 13 February 2024).

#### 2.6.2. Molecular Docking of Potential BH3-Mimetic Peptides

Peptides were predicted to have potential anticancer properties using ACPred^®^ software (https://bio.tools/ACPred, accessed on 17 January 2024). The crystallographic structure of Bcl-2 (PDB: 6O0k) was obtained from the PDB server (https://www.rcsb.org, accessed on 17 February 2024). The protein and ligands (Venetoclax/peptides) were prepared using AutoDock tools^®^ version 1.5.6 (Molecular Graphics Laboratory, The Scripps Research Institute, La Jolla, CA, USA). Water and ligand were extracted from the protein structure. Subsequently, hydrogen atoms and Gasteiger partial charges were incorporated into the carbon atoms that were associated with hydrogen. The structure of the peptides was constructed in a two-dimensional format utilizing Marvin Sketch software (https://marvinjs-demo.chemaxon.com/latest/demo.html, accessed on 20 February 2024) and subsequently preserved in a three-dimensional configuration. The peptides were subsequently optimized to fix the charge, add hydrogen atoms, and minimize energy levels.

The peptides (ligand–receptor affinity) with affinity energy of −170 kcal/mol to the Bcl-2 region of the Venetoclax receptor (Ala100, Gly145, Val148, Asp103, Phe104, Tyr202, Ala149, Tyr108, Met115, Phe112, Val156) were selected using HPEPDOCK software (http://huanglab.phys.hust.edu.cn/hpepdock, accessed on 1 March 2024) [30].

Afterward, the predicted interaction profile of the peptides was compared to the Venetoclax binding site using AutoDock-VINA^®^ software (version 1.1.2) [31] with three replicates for each molecule. The search space for the Bcl-2 structure was set to −15.282(x), 2.238(y), −9.432(z) and 40Å(x), 66Å(y), 40Å(z) for the center and dimensions of the box search, respectively. Ligand interactions were observed using Discovery Biovia^®^ [32] and visualized on PyMOL [33].

#### 2.6.3. Secondary Structure Prediction

The secondary structure of the peptides was predicted using PEP-FOLD4^®^ software [34] (http://bioserv.rpbs.univ-paris-diderot.fr/services/PEP-FOLD4, accessed on 26 March 2024).

#### 2.6.4. Pharmacokinetic Properties

Pharmacokinetic capacities such as human intestinal absorption (HIA); bioavailability (F50%); protein plasma binding (PPB); volume distribution (VDss); blood–brain barrier penetration (BBB); CYP1A2, CYP2C9, and CYP2D6 substrates; clearance (CLplasma); half-life (T_1/2_); and human hepatotoxicity, nephrotoxicity, and neurotoxicity were evaluated using ADMETlab^®^ 3.0 software [35] (https://admetlab3.scbdd.com/, accessed on 26 March 2024).

### 2.7. Statistical Analysis

The cell viability assays were carried out in triplicate; the results are expressed as the arithmetic average ± standard deviation. Significant differences were assessed using one-way analysis of variance (ANOVA), and Tukey’s multiple range test was applied (*p* < 0.05), using GraphPad Prism 10 software (GraphPad Software, Boston, MA, USA).

## 3. Results

### 3.1. Isolation of β-Conglycinin and β-Vignin Proteins

**Figure 1:**
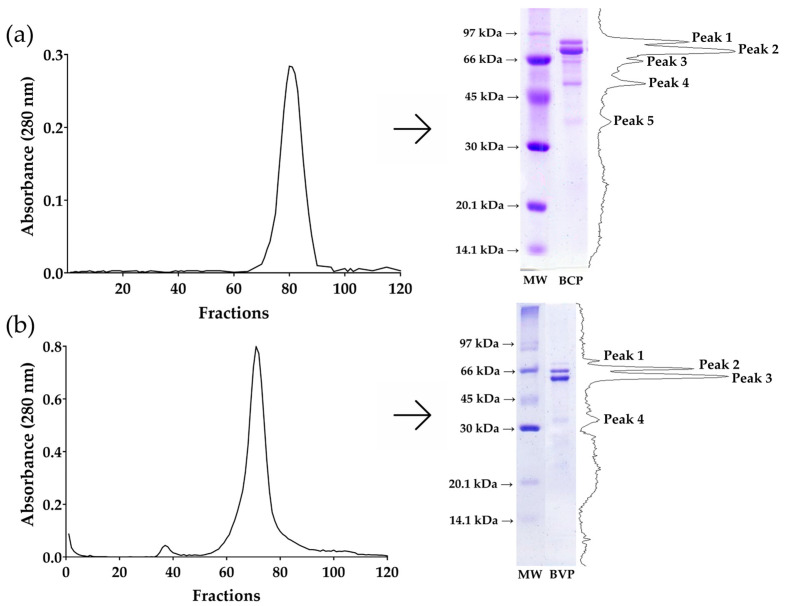
Size exclusion chromatography and SDS-PAGE profile under reducing conditions of (**a**) β-conglycinin from soybean and (**b**) β-vignin from cowpea bean. MW: molecular weight ladder. BCP: β-conglycinin protein. BVP: β-vignin protein.

Figure 1 shows the size exclusion chromatography profile, SDS-PAGE profile under reducing conditions, and densitometric analyses of β-conglycinin from soybean and β-vignin from cowpea bean proteins.

In Figure 1a, the size exclusion chromatography profile of the isolated β-conglycinin from soybean indicates the presence of one peak. The SDS-PAGE of that peak shows five bands (75.4 kDa, 68.6 kDa, 62.8 kDa, 52.3 kDa, and 37.9 kDa), as confirmed by the densitometric analysis. In Figure 1b, the size exclusion chromatography profile of the isolated β-vignin from cowpea bean indicates the presence of one peak. The SDS-PAGE of that peak shows four bands (69.6 kDa, 63.6 kDa, 58.4 kDa, and 37.7 kDa), as confirmed by the densitometric analysis.

### 3.2. Viability of Non-Cancer and Cancer Cells

**Figure 2:**
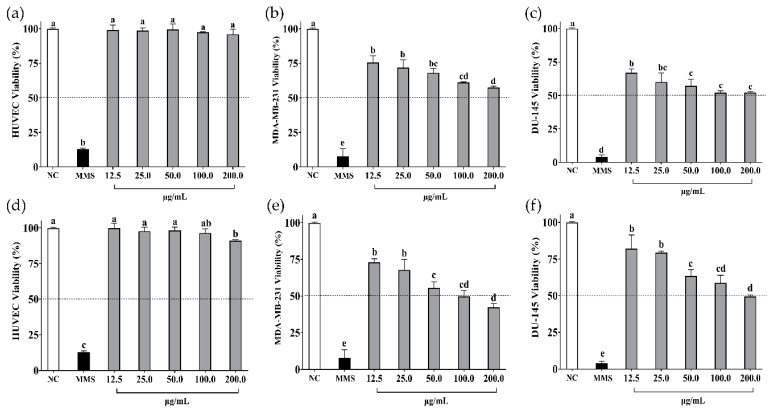
Cell viability (%) of (**a**) HUVEC, (**b**) MDA-MB-231, and (**c**) DU-145 cells when treated with β-conglycinin hydrolysate from soybean. Cell viability (%) of (**d**) HUVEC, (**e**) MDA-MB-231, and (**f**) DU-145 cells when treated with peptides < 3 kDa from β-conglycinin hydrolysate from soybean. NC: negative control—cells not treated. MMS: methyl methane sulfonate at 300 µM (positive control). Data are presented as mean ± standard deviation (*n* = 3). Different letters indicate a difference between concentrations (*p* value < 0.05 according to Tukey’s multiple-range test).

**Figure 3:**
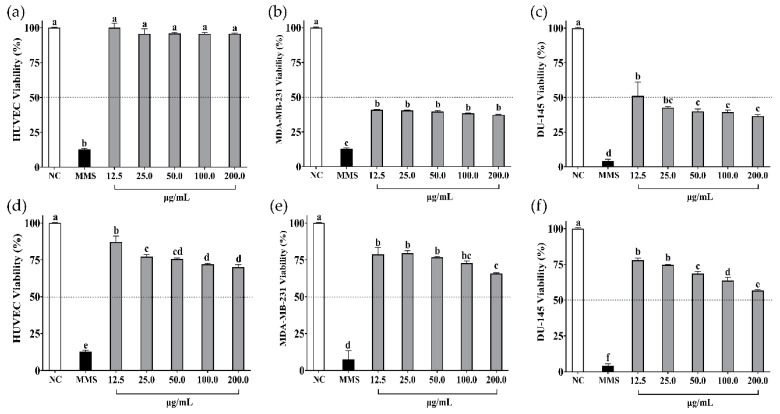
Cell viability (%) of (**a**) HUVEC, (**b**) MDA-MB-231, and (**c**) DU-145 cells when treated with β-vignin hydrolysate from cowpea bean. Cell viability (%) of (**d**) HUVEC, (**e**) MDA-MB-231, and (**f**) DU-145 cells when treated with peptides < 3 kDa from β-vignin hydrolysate from cowpea bean. NC: negative control—cells not treated. MMS: methyl methane sulfonate at 300 µM (positive control). Data are presented as mean ± standard deviation (*n* = 3). Different letters indicate differences between treatments (*p*-value < 0.05 according to Tukey’s multiple-range test).

The in vitro cell viability assays of soybean and cowpea hydrolysates and peptides < 3 kDa on non-cancer cells (HUVEC) and cancer cells (MDA-MB-231, DU-145) are presented in Figure 2 and Figure 3, respectively.

β-conglycinin hydrolysate did not affect the viability of non-cancer cells (*p* > 0.05) (Figure 2a) but did affect cancer cells in a dose-dependent manner (*p* < 0.05) (Figure 2b,c). Peptides < 3 kDa slightly affected the viability of HUVEC cells (9%) at 200 µg/mL (Figure 2d) but had a significant impact on MDA-MB-231 cells (IC_50_ = 97.70 ± 0.10 µg/mL) (Figure 2e), followed by DU-145 cells (IC_50_ = 179.90 ± 0.12 µg/mL) (Figure 2f), in a dose-dependent manner.

β-vignin hydrolysate did not affect non-cancer cells (*p* > 0.05) (Figure 3a); however, it affected cancer cells. Although viability was compromised on MDA-MB-231 and DU-145 cells, it was not dose-dependent (Figure 3b,c). Peptides < 3 kDa from β-vignin reduced the viability of all cells (Figure 3d–f). The viability of MDA-MB-231 cells was not affected in a dose-dependent manner (Figure 3e) by the peptides, unlike the effect observed on DU-145 cells (Figure 3f).

### 3.3. Simulated Gastrointestinal Digestion In Silico

**Figure 4:**
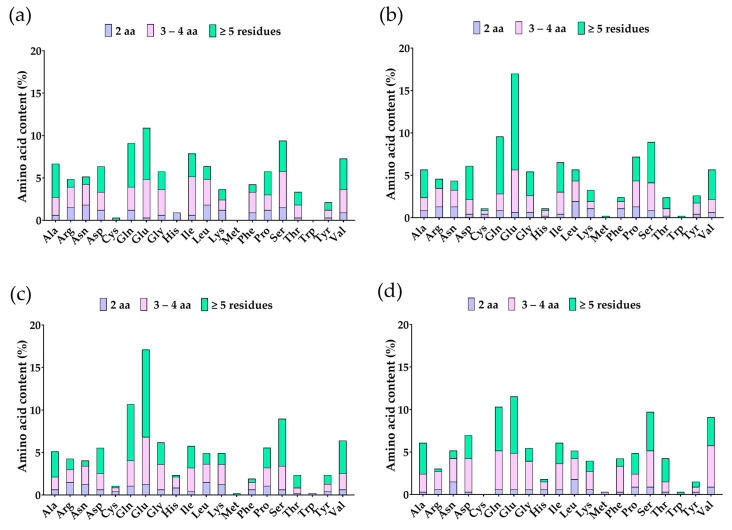
Amino acid content (%) of peptides from soybean β-conglycinin (**a**) β subunit, (**b**) α subunit, (**c**) α’ subunit, and (**d**) cowpea β-vignin from in silico hydrolysis.

Enzymatic hydrolysis with pepsin, trypsin, and chymotrypsin of the β-conglycinin β, α, α’ subunits and β-vignin was simulated through BIOPEP^®^ and released 90, 112, 118, and 84 peptides, respectively. The most abundant amino acids are glutamic acid (Glu), glutamine (Gln), serine (Ser), and valine (Val), mainly in peptides with ≥5 residues (Figure 4). Anticancer peptides were predicted using ACPred^®^, resulting in 48 possible candidate anticancer peptides (Supplementary Table S2).

### 3.4. Potential BH3-Mimetic Effect of Peptides

**Figure 5:**
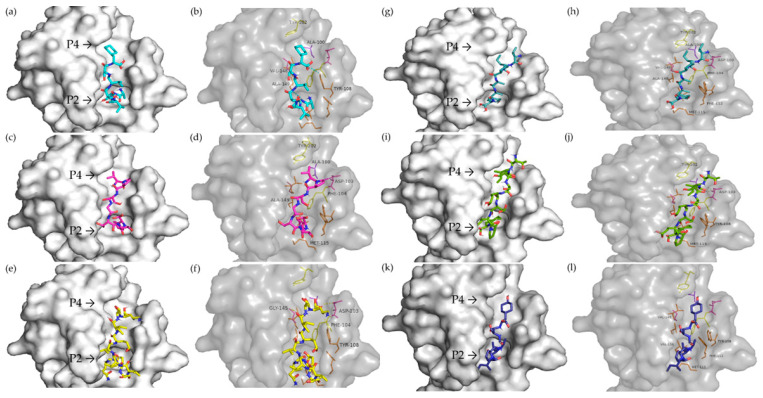
Molecular docking of vicilin-derived peptides on Bcl-2. (**a**) ASVSVSF on the surface of the binding site; (**b**) interactions of ASVSVSF on the BH3 binding site; (**c**) VPSGTTY on the surface of the binding site; (**d**) interactions of VPSGTTY on the BH3 binding site; (**e**) QESVIVEISK on the surface of the binding site; (**f**) interactions of QESVIVEISK on the BH3 binding site; (**g**) VIPAAY on the surface of the binding site; (**h**) interactions of VIPAAY on the BH3 binding site; (**i**) TISSEDEPF on the surface of the binding site; (**j**) interactions of TISSEDEPF on the BH3 binding site; (**k**) VIPASY on the surface of the binding site; (**l**) interactions of VIPASY on the BH3 binding site. The residues highlighted and labeled in the pictures are the same amino acids that Venetoclax interacts with Bcl-2.

Six peptides could interact with Bcl-2 at the BH3 site (Figure 5). ASVSVSF peptide was predicted to be able to interact with the P2 and P4 pockets (Figure 5a). Alkyl-type binding with Ala100 and Val148, Pi-alkyl with Tyr 202, and conventional hydrogen bonding with Tyr108 in the P4 pocket were observed, while in the P2 pocket, interactions occurred through a conventional hydrogen bond with Ala149 and an attractive charge bond with Glu152 (Figure 5b).

The VPSGTTY peptide interacted with Asp103 via a carbon–hydrogen bond, with Ala100 through an alkyl bond, and with Tyr202 via a Pi-Sigma bond in the P4 pocket. Conventional hydrogen bonds with Asn143, Leu137, and Arg146 occurred between P2 and P4. Only hydrophobic interactions occurred in P2 with Ala149, Met115, and Phe104 (Figure 5c,d).

The QESVIVEISK peptide, on the other hand, interacted with the binding site in both pockets via a hydrogen bond with Tyr108, Asp103, Phe104, Gly145, Asn143, and Asp111 (Figure 5e,f).

VIPAAY displayed more similar interactions with Venetoclax-binding residues than any other peptide. Carbon–hydrogen bonds with Phe112 and alkyl-type bonds with Met115, Phe104, and Ala149 occurred in the P2 pocket. A conventional hydrogen bond occurred with Asn143, alkyl with Val148 and Ala100, Pi-Sigma with Tyr202, and salt bridge with Asp103 in the P4 pocket (Figure 5g,h).

The TISSEDEPF peptide displayed an alkyl bond with Met115 and a Pi-sigma bond with Tyr108 in the P2 pocket. Several interactions, mostly in the P4 pocket, occurred through a conventional hydrogen bond with Glu136, Leu137, Asn143, Tyr202, Asp103, and Arg107 (Figure 5i,j).

The VIPASY peptide bonded with Asp111 via an attractive charge interaction; with Val133, Val156, and Met115 via alkyl interaction; and with Phe112 through a Pi-alkyl bond in the P2 pocket. Between pockets, a Pi-sigma bond was observed with Tyr108. And an alkyl bond occurred in the P4 pocket with Val148 (Figure 5k,l).

**Figure 6:**
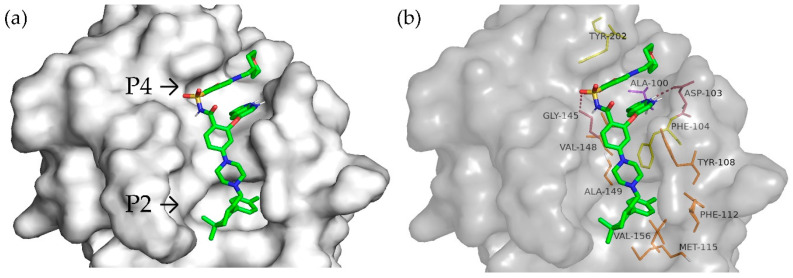
Molecular docking of Venetoclax on Bcl-2. (**a**) Venetoclax positioning on the surface of the binding site; (**b**) interactions of Venetoclax with amino acid residues of the BH3 binding site.

The molecular docking of Venetoclax, a well-known targeted drug acting as a cancer growth blocker by inhibiting Bcl-2, was also performed to compare and validate the above-reported predictions. The results show (Figure 6) that it occupied the P2 and P4 pockets of Bcl-2 (Figure 6a). A conventional hydrogen bond was formed with Gly145 and Asp103 in the P4 pocket, and hydrophobic interactions occurred, such as Pi–Pi stacked interacted with Tyr202, Pi–Pi T-shaped with Phe104, and alkyl with Ala100 and Val148. In the P2 pocket, alkyl and Pi-alkyl bonds with Ala149, Tyr108, Met115, Phe112, and Val156 were observed (Figure 6b). The interactions of peptides and Venetoclax with the BH3 site are summarized in Supplementary Table S3.

### 3.5. Secondary Structure of Potential BH3-Mimetic Vicilin-Derived Peptides

**Figure 7:**
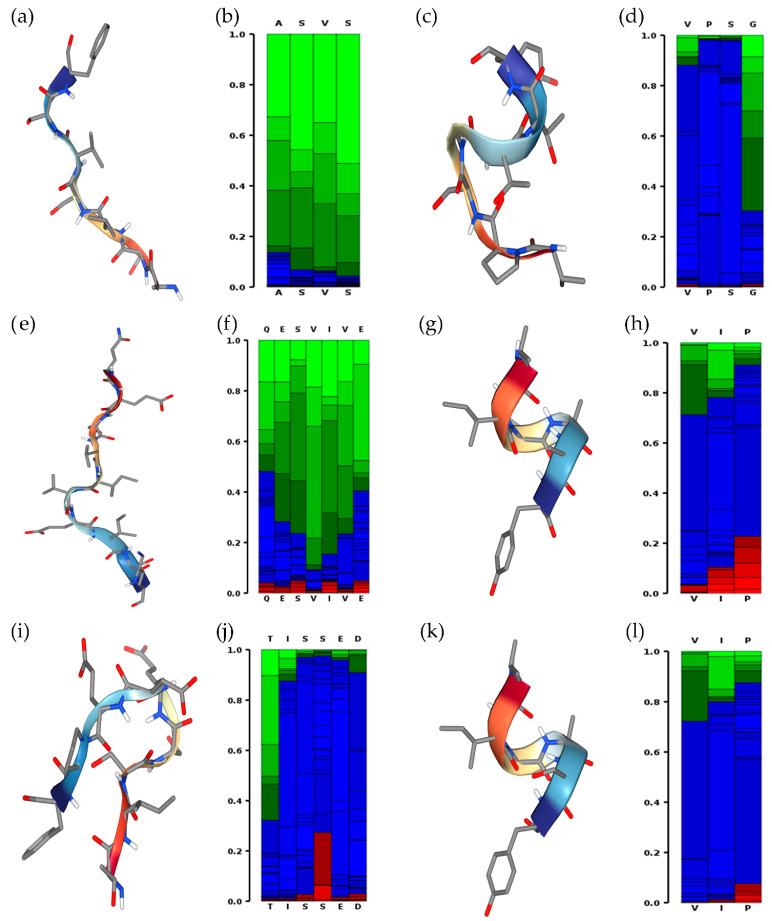
Secondary structure of (**a**,**b**) ASVSVSF, (**c**,**d**) VPSGTTY, (**e**,**f**) QESVIVEISK, (**g**,**h**) VIPAAY, (**i**,**j**) TISSEDEPF, and (**k**,**l**) VIPASY peptides predicted on PEPFOLD4^®^. Red, green, and blue colors indicate helical, extended, or other state propensities, respectively.

The prediction of the secondary structure of ASVSVSF, VPSGTTY, QESVIVEISK, VIPAAY, TISSEDEPF, and VIPASY peptides is illustrated in Figure 7. VIPAAY and VIPASY peptides were the only ones that displayed a visual helical state as a secondary structure.

### 3.6. Pharmacokinetic and Toxicologic Features

The ADMET properties of Venetoclax and vicilin-derived peptides are illustrated in Table 1. Venetoclax, VPSGTTY, VIPAAY, and VIPASY peptides scored very well for HIA. Venetoclax was excellent, but all peptides showed poor F50%. Regarding the distribution parameters, only the peptides scored very well for PPB. All compounds showed poor VDss and excellent BBB. Metabolism prediction indicated that Venetoclax is a substrate for CYP3A4. For excretion, CLplasma scored well for all compounds; however, T1/2 scored poorly for Venetoclax and the VIPAAY peptide. Regarding toxicity, they were all nephrotoxic, and only Venetoclax was hepatotoxic. However, ASVSVSF, QESVIVEISK, VIPAAY, and VIPASY showed some neurotoxicity.

## 4. Discussion

Vicilins are a good source of bioactive peptides [36,37]. Previous studies have shown that β-conglycinin from soybean may inhibit colon cancer cells (HCT-116) [38], and its hydrolysate inhibits leukemia cell (L1210) growth in vitro [39]. Additionally, cowpea seed protein hydrolysate presents antioxidant activity and inhibition of ACE [37], and its β-vignin protein is a good source of hypocholesterolemic peptides [8,9,10]. β-vignin, the vicilin-like protein of cowpea, consists of three subunits ranging from 40 kDa to 75 kDa [40]. The cleavage of its subunits into smaller molecules produces bioactive peptides [41]. Our densitometric analyses of isolated proteins confirmed the identity of β-conglycinin from soybean and β-vignin from cowpea bean. In Figure 1a, the major bands correspond to the α’ subunit (peak 1), α subunit (peak 2), and β subunit (peak 4) of β-conglycinin, consistent with previous work [22,42]. In Figure 1b, the major polypeptides (peak 2 and 3) correspond to vicilin subunits from cowpea bean [40,43].

Then, β-conglycinin and β-vignin proteins were hydrolyzed with pepsin and pancreatin enzymes, which, as expected [44], successfully generated legume-derived peptides with anticancer properties (Figure 2 and Figure 3). Evidence shows that food-derived anticancer peptides consist of 3–25 amino acid residues [45] and peptide-based antitumor agents exhibit low molecular weight [46]. In previous studies, the fraction consisting of peptides < 5 kDa exerted the most promising anticancer effect compared with larger peptides in breast cancer cells (MCF-7) [47] and colorectal cancer cells (HCT-116 and RKO) [48]. In the present study, peptides < 3 kDa from both hydrolysates were separated for further analysis.

In the cell viability tests, biological effects were observed on breast and prostate cancer cells but not on the non-cancer ones. β-conglycinin hydrolysates showed a dose-dependent effect not observed when cancer cells were treated with β-vignin hydrolysates. On the other hand, a dose-dependent viability decrease was observed for peptides < 3 kDa from both β-conglycinin and β-vignin. Their dose-dependent nature can be influenced by the specific characteristics of each cell type [49], dose, and time of treatment [50]. Additionally, interactions between compounds can alter expected dose-dependent outcomes [51], which could have happened considering hydrolysates comprise several peptides. Furthermore, possible antagonistic or synergistic effects cannot be excluded, especially for fractions whose composition is more complex, such as hydrolysates. It is important to point out that because the performed experimental replicas are limited, the cell viability assays should be considered inconclusive.

Overall, these findings corroborate several studies showing that shorter peptides from protein hydrolysates usually exert more significant anticancer activity than larger peptides [48,52]. They also present higher mobility and diffusivity than higher-weight peptides, interacting more effectively with cancer cells [46]. Peptides < 4 kDa from black soybean showed a significant (*p* < 0.05) antiproliferative effect on MCF-7 breast cancer cells compared to higher-molecular-weight peptides (4–6 kDa and > 6 kDa fractions), with an IC_50_ of 276 µg/mL [47], higher than treatment with peptides < 3 kDa from β-conglycinin observed in the present study. The non-digestible fraction of common bean (*Phaseolus vulgaris* L.) hydrolyzed with pepsin/pancreatin presented antiproliferative activity in colorectal cancer cells (HCT-116 and RKO). Five peptides (GLTSK, LSGNK, GEGSGA, MPACGSS, and MTEEY) with molecular masses ranging from 505 to 1019 Da were the most abundant in the hydrolysate [52]. GLTSK (IC_50_ = 134.6 μM) and GEGSGA (IC_50_ = 156.7 μM) peptides were able to inhibit colon cancer (HCT-116) cell growth in a dose–response manner, interacted synergistically with oxaliplatin, triggered cell cycle arrest (G2 and G1), and induced apoptosis (32.9% and 23.7%) by causing loss of mitochondrial membrane potential (16.1% and 7.4%), releasing proapoptotic signals (such as Bax), and increasing intracellular ROS concentration, respectively [48].

Bioinformatic predictions can be considered efficient, cost-effective, and time-saving tools for driving and addressing further research, starting from experimental data. Despite its inherent limitations, this approach has been successfully used to screen potential peptide sequences with biological effects from different food origins [53]. Using traditional methods to identify possible anticancer peptides would require high-throughput screenings. ACPred is a tool aimed at predicting peptides with anticancer activity, achieving a successful prediction rate of up to 95.61% through a web server interface [54]. Similarly, the HPEPDOCK tool is available as a web server and outperforms its competitors, such as the pepATTRACT server and HADDOCK [55]. While these in silico approaches do not prove the biological activity of peptides, they are helpful for prioritizing peptides for experimental evaluation [53]. For these reasons, we used in silico tools to predict candidates and identified the peptides likely released from β-conglycinin and β-vignin proteins, with anticancer activity based on their interactions with the BH3 domain of Bcl-2. Protein databases contain several full-size vicilin-like proteins from soybeans, whereas only one sequence of vicilin protein was found for cowpea beans. This explains why we could retrieve more soybean-derived peptide sequences by querying the databases. The availability of sequences permits the inference of substantial information about the amino acid profiles of the identified peptides. The physical–chemical characteristics of the single amino acids of peptides could be associated with anticancer features, especially the presence of Glu, Ser, Pro, Leu, Gly, Ala, Lys, Arg, Thr, and Tyr [45], predominantly hydrophobic residues. Peptides ≥ 5 residues from our in silico hydrolysis had more hydrophobic residues, such as Glu, Ser, Val, and Gln, leading the investigation of this group of peptides.

Of the 48 peptides predicted by ACPred^®^ to have anticancer potential, 6 showed good BH3-binding scores, as shown by the molecular docking analysis. VIPAAY from the soybean β-conglycinin β subunit (amino acid sequence n. 95 in Supplementary Table S3) is the peptide that most closely mimics Venetoclax’s binding behavior. It binds to eight key residues within the P2 and P4 pockets of Bcl-2, specifically Phe112, Met115, Phe104, Ala149, Val148, Ala100, Tyr202, and Asp103. This unique interaction profile distinguishes VIPAAY from other peptides in this study. Venetoclax is one of the first approved drugs that selectively targets Bcl-2 [56] and, thus, presents reduced harmful side effects compared to BH3-mimetics that non-specifically target multiple Bcl-2 family proteins [57]. Hence, the more similar interactions a new compound exhibits at the BH3-Venetoclax binding site, the higher the chance that the compound must function as a Bcl-2 inhibitor.

Structural predictions of the six peptides showed that VIPAAY and VIPASY (peptide n. 13 in Supplementary Table S3) present a potential helical secondary structure, which is a crucial feature for selecting anticancer candidates and designing therapeutic compounds [58]. Research indicates that helical conformation enhances the peptides’ ability to disrupt cancer cell membranes selectively, a mechanism that is less likely to affect normal cells [59]. Additionally, the stability conferred by the helical structure is essential for maintaining the functional integrity of peptides in the physiological environment [60].

Another characteristic to be considered in the search for new therapeutic compounds is their ADME/T properties. The importance of ADME/T properties relies on comprehending their impact on pharmacokinetic and pharmacodynamic outcomes for new drug candidates. A compound must be satisfactorily absorbed and distributed to reach its target site without being prematurely metabolized or excreted. Moreover, metabolism is involved in the biotransformation, affecting compounds’ activity and potential toxicity [61].

The oral route of drug delivery offers numerous benefits in comparison to alternative administration routes, making it the most common [62]. It is generally more convenient, less invasive than parenteral routes, and cost-effective, and allows for a wide range of formulations. Likewise, it can provide a controlled release, which helps maintain steady drug levels in the bloodstream and minimizes side effects associated with peak concentrations [63]. Venetoclax showed excellent human intestinal absorption (HIA) and bioavailability (F_50%_), as it is administered orally [64]. The absorption and bioavailability of Venetoclax are mainly influenced by food intake since administration with food maximizes its absorption and therapeutic effect, considering it is a highly hydrophobic drug [65]. VPSGTTY, VIPAAY, and VIPASY peptides showed good HIA potentials as predicted, but all peptides presented poor F_50%_. This may be explained by the fact that serum peptidases could degrade peptides and, therefore, are less available in systemic circulation [66]. Regarding distribution, all peptides are indicated to have excellent protein plasma binding (PPB), better than Venetoclax. This is an interesting indicator considering that PPB is the principal determinant of tissue distribution and receptor interaction, and it is also associated with drug safety and adverse side effects [67]. The peptides are not substrates for CYP1A2, CYP2C9, and CYP2D6, major catalysts involved in most drug oxidations [68]; therefore, PPB could be beneficial as it may reduce the complexity of dosing and minimize potential interactions. All peptides, except VIPAAY, scored better than Venetoclax regarding half-life (T_1/2_). Venetoclax presents a terminal phase elimination half-life (t1/2) of approximately 18 h [69], which may be explained by its high protein-bound capacity (PPB) of >90%. Since the calculated PPB for the peptides was lower than 90%, this elucidates the rationale behind their shorter half-life (t1/2). All peptides may, therefore, be less hepatotoxic and neurotoxic than Venetoclax, indicating that they may be safer and cause fewer side effects than this drug.

It is worth noting that the methodological approach of this study presents certain limitations. For example, the non-cancer cells used in the study (HUVEC) were not from the same organ as the cancer cells (MDA-MB-231 and DU-145). Nonetheless, the results offer significant contributions and novel perspectives. In addition, we are aware that although several validated computational tools are substantial assets for screening potential anticancer peptides, experimental tests of the predicted peptides will be required to confirm their predicted BH3-mimetic effect.

## 5. Conclusions

Our study experimentally demonstrated that <3 kDa peptides from soybean β-conglycinin and cowpea β-vignin proteins could inhibit breast and prostate cancer cells in vitro, in a dose-dependent manner. The in silico analyses indicated that six potential BH3-mimetic peptides may be released from the parental proteins. In particular, the VIPAAY peptide from β-conglycinin most closely mimics Venetoclax’s binding behavior. The prediction of its secondary structure as having alpha-helix and ADMET/T properties reinforced the possibility of it presenting anticancer activity. Experiments using VIPAAY synthetic peptides (and possibly the other five identified sequences) in various cell models, including those not tested in this work, are necessary to gain further insight and provide experimental evidence to confirm the potentiality of VIPAAY until the mechanisms of action at the molecular level are unveiled.

## Figures and Tables

**Table 1:** ADMET properties of Venetoclax and vicilin-derived peptides.

Compound	VIPASY		ASVSVSF		VPSGTTY		QESVIVEISK		VIPAAY		TISSEDEPF		Venetoclax	
Absorption														
HIA ^a^	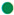	0.002	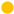	0.581	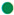	0.016	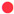	1.000	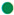	0.001	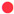	1.000	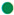	0.000
F_50%_^b^	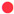	1.000	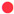	1.000	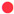	1.000	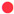	1.000	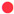	1.000	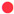	1.000	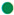	0.001
Distribution														
PPB ^c^	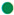	54.410	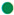	34.033	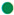	37.469	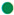	1.148	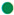	62.698	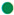	33.091	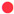	99.575
VDss ^d^	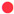	−0.519	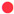	−0.430	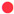	−0.526	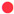	−0.733	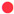	−0.620	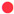	−0.641	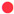	−0.008
BBB ^e^	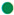	0.000	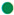	0.000	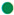	0.000	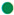	0.000	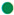	0.000	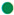	0.000	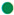	0.005
Metabolism														
CYP1A2 Substrate ^f^	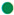	0.000	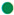	0.000	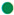	0.000	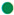	0.000	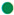	0.000	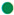	0.000	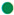	0.029
CYP3A4 Substrate ^f^	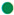	0.000	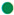	0.000	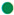	0.000	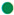	0.000	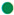	0.001	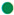	0.000	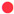	1.000
CYP2D6 Substrate ^f^	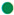	0.000	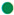	0.000	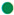	0.000	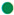	0.000	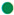	0.000	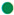	0.000	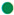	0.004
Excretion														
CL_plasma_^g^	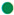	2.387	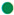	1.993	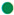	1.512	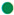	1.660	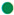	2.092	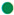	1.532	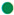	2.298
T_1/2_^h^	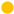	1.000	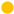	1.474	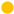	1.319	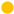	1.376	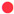	0.943	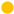	1.356	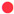	0.671
Toxicity														
Hepatotoxicity ^i^	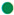	0.209	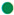	0.006	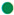	0.113	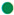	0.009	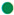	0.230	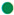	0.084	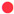	0.910
Nephrotoxicity ^j^	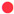	0.954	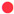	0.993	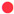	0.979	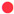	0.998	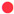	0.908	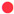	1.000	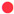	0.937
Neurotoxicity ^k^	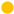	0.477	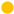	0.523	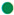	0.006	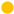	0.367	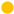	0.633	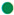	0.001	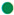	0.122

The three colored labels represent the following: 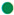: excellent; 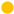: medium; 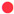: poor. ^a^ HIA: human intestinal absorption → Category 1: HIA+ (HIA < 30%); Category 0: HIA-(HIA ≥ 30%). The output value is the probability of being HIA+. ^b^ F_50%_: 50% bioavailability; Category 1: F 50% + (bioavailability < 50%); Category 0: F 50% (bioavailability ≥ 50%). The output value is the probability of being F 50% +. ^c^ PPB: protein plasma binding. Optimal: <90%. Drugs with high protein binding may have a low therapeutic index. ^d^ VDss: volume distribution. Optimal: 0.04–20 L/kg. ^e^ BBB: blood–brain barrier penetration. Category 1: BBB+; Category 0: BBB−. The output value is the probability of being BBB+. ^f^ CYP1A2, CYP2C9, and CYP2D6 substrates—Category 1: substrate; Category 0: non-substrate. The output value is the probability of being substrate. ^g^ CL_plasma_: clearance. The unit of predicted CLplasma penetration is ml/min/kg. >15 mL/min/kg: high clearance; 5–15 mL/min/kg: moderate clearance; <5 mL/min/kg: low clearance. ^h^ T_1/2_: half-life. The unit of predicted T1/2 is hours. Ultra-short half-life drugs: T1/2 < 1 h; short half-life drugs: T1/2 between 1 and 4 h; intermediate short half-life drugs: T1/2 between 4 and 8 h; long half-life drugs: T1/2 > 8 h. ^i^ Human hepatotoxicity: Category 1: H-HT positive (+); Category 0: H-HT negative (−). The output value is the probability of being toxic. ^j^ Drug-induced nephrotoxicity: Category 0: non-nephrotoxic (−); Category 1: nephrotoxic (+). The output value is the probability of being nephrotoxic (+), within the range of 0 to 1. ^k^ Drug-induced-neurotoxicity: Category 0: non-neurotoxic (−); Category 1: neurotoxic (+). The output value is the probability of being neurotoxic (+), within the range of 0 to 1.

## Data Availability

All methods and related data are presented in this paper. Additional inquiries should be addressed to the corresponding author.

## Supplementary Materials

The following supporting information can be downloaded at https://www.mdpi.com/article/10.3390/foods13213508/s1: Table S1: Vicilin proteins from soybean (*Glycine max*) and cowpea bean (*Vigna unguiculata*) listed at Uniprot^®^ databank. Table S2: Prediction of anticancer peptides according to ACPred^®^. Table S3: Docking predictions of selected peptides and Venetoclax interactions with the BH3 site of Bcl-2.
