# Supplementary material for: Soybean β-Conglycinin and Cowpea β-Vignin Peptides Inhibit Breast and Prostate Cancer Cell Growth: An In Silico and In Vitro Approach
Source: Foods. 2024 Nov 1;13(21):3508. doi: 10.3390/foods13213508 (PMC11545662; doi:10.3390/foods13213508)
Supplement: Supplementary file 1 [file foods-13-03508-s001.zip › foods-3226279-supplementary.pdf]

foods

MDPI

Table S1. Vicilin proteins from soybean (*Glycine max*) and cowpea bean (*Vigna unguiculata*) listed at Uniprot® databank.

Source	Protein	Uniprot ID	Nº of Amino Acids	Protein Size (kDa)
Glycine max	β-conglycinin β subunit 1	P25974	439	50.47
Glycine max	β-conglycinin β subunit 2	F7J077	439	50.44
Glycine max	β-conglycinin α subunit 1	P0DO16	605	70.31
Glycine max	β-conglycinin α subunit 2	P0DO15	605	70.31
Glycine max	β-conglycinin α' subunit	P11827	621	72.23
Vigna unguiculata	β-vignin	A8YQH5	433	49.69

Table S2. Prediction of anticancer peptides according to ACPred®.

Specie	Protein	Nº	Peptide	ACP	Non-ACP	Prediction
Cowpea bean	β-vignin	1	ADADF	0.946	0.054	ACP
		2	AVPVN	0.182	0.818	non-ACP
		3	AIVIL	0.944	0.056	ACP
		4	QSDSH	0.859	0.141	ACP
		5	QESQEESEPR	0.671	0.329	ACP
		6	SSTEAQQSY	0.461	0.539	non-ACP

Table S2. Cont.

Specie	Protein	Nº	Peptide	ACP	Non-ACP	Prediction
Cowpea bean	β-vignin	7	QQDEESQQEGVIVQL	0.072	0.928	non-ACP
		8	GEEEQK	0.910	0.090	ACP
		9	EITPEK	0.836	0.164	ACP
		10	TSVDIK	0.923	0.077	ACP
		11	EQQQQQQEESW	0.841	0.159	ACP
		12	AEVSDDDVF	0.384	0.616	non-ACP
		13	VIPASY	0.918	0.082	ACP
		14	PVAITATSN	0.124	0.876	non-ACP
		15	PASGEK	0.269	0.731	non-ACP
		16	AGEEDN	0.594	0.406	non-ACP
		17	SEIPTEVL	0.772	0.228	ACP
Soybean	β-conglycinin α subunit	18	QEEEH	0.956	0.044	ACP
		19	EEQEW	0.969	0.031	ACP
		20	ADADY	0.939	0.061	ACP
		21	GTAIL	0.801	0.199	ACP
		22	AIPVN	0.182	0.818	non-ACP
		23	SIVDM	0.829	0.171	ACP
		24	AIVIL	0.944	0.056	ACP

	Nº	Peptide	ACP	Non-ACP	Prediction
	25	EGDAN	0.164	0.836	non-ACP
	26	PVVVN	0.853	0.147	ACP
	27	AIGIN	0.907	0.093	ACP
	28	EECEEGEIPR	0.890	0.110	ACP
	29	VDAQPK	0.409	0.591	non-ACP
	30	GSEEEDEDEDEEQDER	0.963	0.037	ACP
	31	ASVSVSF	0.968	0.032	ACP
	32	EPQQPGEK	0.151	0.849	non-ACP
	33	EEDEDEQPR	0.941	0.059	ACP
	34	QEEDEDEEQQR	0.941	0.059	ACP
	35	ESEESEDSEL	0.931	0.069	ACP
	36	QSGDAL	0.023	0.977	non-ACP
	37	VPSGTTY	0.785	0.215	ACP
	38	SSTEAQQSY	0.461	0.539	non-ACP
	39	EEGQQQGEQR	0.449	0.551	non-ACP
	40	QESVIVEISK	0.908	0.092	ACP
	41	TISSEDK	0.720	0.280	ACP
	42	EITPEK	0.836	0.164	ACP
	43	EQQQEQQQEEQPL	0.769	0.231	ACP
	44	SEQDIF	0.824	0.176	ACP
	45	VIPAGY	0.932	0.068	ACP

Table S2. Cont.

	Nº	Peptide	ACP	Non-ACP	Prediction
	46	AGSQDN	0.015	0.985	non-ACP
	47	VISQIPSQVQEL	0.394	0.606	non-ACP
	48	PGSAQAVEK	0.126	0.874	non-ACP
β-conglycinin α' subunit	49	EQQQR	0.943	0.057	ACP
	50	PGSAK	0.452	0.548	non-ACP
	51	PVVVN	0.853	0.147	ACP
	52	ATSDL	0.267	0.733	non-ACP
	53	AIVVL	0.864	0.136	ACP
	54	SVVDM	0.805	0.195	ACP
	55	EGEAN	0.726	0.274	ACP
	56	GTAIL	0.801	0.199	ACP
	57	QEEEH	0.956	0.044	ACP
	58	ADADY	0.939	0.061	ACP
	59	AIPVN	0.182	0.818	non-ACP
	60	VDAQPQQK	0.053	0.947	non-ACP
	61	VISQIPSQVQEL	0.394	0.606	non-ACP
	62	SEQDIF	0.824	0.176	ACP
	63	SQSESY	0.945	0.055	ACP

	64	VIPAGY	0.932	0.068	ACP
	65	QQQEEQPL	0.517	0.483	non-ACP
	66	TISSEDK	0.720	0.280	ACP
	67	EITPEK	0.836	0.164	ACP
	68	QESVIVEISK	0.908	0.092	ACP
	69	ESEEEEEDQDEDEEQDK	0.964	0.036	ACP
	70	EEGQQQGEER	0.719	0.281	ACP
	71	EEDEGEQPR	0.822	0.178	ACP
	72	VEEEEECEEGQIPR	0.871	0.129	ACP
	73	SSTQAQQSY	0.554	0.446	non-ACP
	74	GSEEEQDER	0.886	0.114	ACP
	75	QSGDAL	0.023	0.977	non-ACP
	76	ASVSVSF	0.968	0.032	ACP
	77	VPAGTTY	0.539	0.461	non-ACP
β-conglycinin β subunit	78	ADADF	0.946	0.054	ACP
	79	AIPVN	0.182	0.818	non-ACP
	80	AIVIL	0.944	0.056	ACP
	81	EGDAN	0.164	0.836	non-ACP
	82	EQQQK	0.946	0.054	ACP
	83	QVQEL	0.471	0.529	non-ACP
	84	EEGSK	0.960	0.040	ACP

Table S2. Cont.

	Nº	Peptide	ACP	Non-ACP	Prediction
	85	ASVCVSL	0.831	0.169	ACP
	86	PGDAQR	0.007	0.993	non-ACP
	87	IPAGTTY	0.850	0.150	ACP
	88	SSTQAQQSY	0.554	0.446	non-ACP
	89	GEEEEQR	0.941	0.059	ACP
	90	QQEGVIVEL	0.367	0.633	non-ACP
	91	TISSEDEPF	0.657	0.343	ACP
	92	EITPEK	0.836	0.164	ACP
	93	SSVDIN	0.615	0.385	non-ACP
	94	SEDDVF	0.918	0.082	ACP
	95	VIPAAY	0.864	0.136	ACP
	96	PGSAQDVER	0.003	0.997	non-ACP
	97	VDAQPQQK	0.053	0.947	non-ACP

Table S3. Interactions by docking predictions of selected peptides and Venetoclax with the BH3 site of Bcl-2.

Ligand	Source	Docking Score (kcal/mol)	Interactions on BH3 Binding Site of Bcl-2
Venetoclax	-	-	Ala100, Gly145, Val148, Asp103, Phe104, Tyr202, Ala149, Tyr108, Met115, Phe112, Val156
ASVSVSF	β-conglycinin *α* and *α′* subunits	-159.399	Tyr108, Ala149, Glu152, Tyr202, Ala100, Val148
VPSGTTY	β-conglycinin *α* subunit	-159.906	Ala149, Met115, Phe104, Leu137, Arg146, Tyr202, Ala100, Asp103, Asn143
QESVIVEISK	β-conglycinin *α* and *α′* subunits	-174.955	Tyr108, Asp103, Phe104, Gly145, Asn143, Asp111
VIPAAY	β-conglycinin β subunit	-158.623	Phe112, Met115, Phe104, Ala149, Asp103, Tyr202, Ala100, Asn143, Val148
TISSEDEPF	β-conglycinin β subunit	-158.491	Met115, Tyr108, Glu136, Leu137, Asn143, Tyr202, Asp103, Arg107
VIPASY	β-vignin	-169.367	Phe112, Met115, Val156, Tyr108, Asp111, Val133, Val148
